# Spin Excitations of High Spin Iron(II) in Metal–Organic Chains on Metal and Superconductor

**DOI:** 10.1002/advs.202412351

**Published:** 2024-12-24

**Authors:** Jung‐Ching Liu, Chao Li, Outhmane Chahib, Xing Wang, Simon Rothenbühler, Robert Häner, Silvio Decurtins, Ulrich Aschauer, Shi‐Xia Liu, Ernst Meyer, Rémy Pawlak

**Affiliations:** ^1^ Department of Physics University of Basel Klingelbergstrasse 82 Basel 4056 Switzerland; ^2^ Department of Chemistry Biochemistry and Pharmaceutical Sciences W. Inäbnit Laboratory for Molecular Quantum Materials University of Bern Freiestrasse 3 Bern 3012 Switzerland; ^3^ Paul Scherrer Institut Forschungsstrasse 111 Villigen PSI 5232 Switzerland; ^4^ Department of Chemistry and Physics of Materials Paris‐Lodron University Salzburg Jakob‐Haringer‐Strasse 2a Salzburg 5020 Austria

**Keywords:** coordination chemistry, magnetocrystalline anisotropy, scanning probe microscopy, spin excitation, superconductivity

## Abstract

Many‐body interactions in metal–organic frameworks (MOFs) are fundamental for emergent quantum physics. Unlike their solution counterpart, magnetization at surfaces in low‐dimensional analogues is strongly influenced by magnetic anisotropy (MA) induced by the substrate and still not well understood. Here, on‐surface coordination chemistry is used to synthesize on Ag(111) and superconducting Pb(111) an iron‐based spin chain by using pyrene‐4,5,9,10‐tetraone (PTO) precursors as ligands. Using low‐temperature scanning probe microscopy, their structures and low‐energy spin excitations of coordinated Fe atoms are compared with high *S* = 2 spin‐state. Although the chain and coordination centers are identical on both substrates, the long‐range spin–spin coupling due to a superexchange through the ligand on Ag is not experimentally observed on Pb(111). This reduction of spin‐spin interactions on Pb in tunneling spectra is ascribed to the depletion of electronic states around the Fermi level of the Pb(111) superconductor as compared to silver.

## Graphical abstract

A spin chain is formed with metal–organic frameworks on surfaces of Ag(111) and Pb(111). Tunneling spectroscopy shows spin‐flip excitations from high spin Fe(II) as well as a long‐range spin–spin coupling on Ag(111), which is absent on Pb(111) due to the depletion of electronic states near the Fermi level.

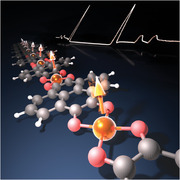

## 1 Introduction

Many‐body interactions inMOFs are at the forefront of emergent quantum physics such as superconductivity,^[^ ^1^, ^2^ ^]^ ferromagnetism^[^ ^3^, ^4^, ^5^, ^6^ ^]^ or quantum spin liquid.^[^ ^7^, ^8^ ^]^ With their high versatility, two‐dimensional metal–organic frameworks (2D MOFs) are prime candidates for inducing such exotic phenomena^[^ ^9^ ^]^ since they use coordination chemistry to associate atomic magnets with multi‐dentate organic ligands at surfaces through self‐assembly processes.^[^ ^10^, ^11^, ^12^ ^]^ 2D MOFs thus allow the precise arrangement of interacting spins into complex lattices and a control over their exchange interaction by judiciously adjusting the inter‐adatom distance. Lattice geometry and adatom spacing over the surface are controlled by the rational design of the organic linkers,^[^ ^10^ ^]^ and their coordination to metal centers, which preferentially show high spin magnetic moments.^[^ ^13^, ^14^ ^]^

In MOFs, magnetic exchange interactions are interpreted in terms of superexchange interactions through the organic ligands.^[^ ^14^, ^15^, ^16^ ^]^ At surfaces, surface‐induced MA forces individual magnetic moments in 2D MOFs to orient along preferential directions, while electronic overlap between these local moments through the metal electrons in the regime of the Ruderman–Kittel–Kasuya–Yosida (RKKY) exchange^[^ ^17^, ^18^, ^19^ ^]^ can also lead to complex magnetic phases.^[^ ^16^ ^]^ These effects can be seen as an opportunity to tune the 2D MOF magnetism not only by considering MA^[^ ^13^, ^20^ ^]^ and interatomic coupling^[^ ^21^ ^]^ but also the intrinsic band structure of the underlying substrate. For instance, superconductors have shown to protect excited spin states^[^ ^22^, ^23^ ^]^ due to opening of the energy gap Δ around the Fermi level *E*_F_ when the condensation of electrons into Cooper pairs occurs at low temperatures. RKKY interaction between magnetic atoms on superconductors^[^ ^24^ ^]^ has also been shown to play a pivotal role for the experimental realization of topological superconductivity.^[^ ^25^, ^26^, ^27^ ^]^ In this context, extending the synthesis of 2D MOFs on superconductors^[^ ^28^, ^29^ ^]^ is of paramount importance in engineering designer quantum materials, topological superconductors or protecting excited spin states in spin logic devices.^[^ ^22^, ^23^ ^]^

To characterize spin excitations at the atomic level, scanning tunneling microscopy (STM) and spectroscopy (STS) are particularly suitable techniques since they allow to probe the local density of states (LDOS) and spin states between tip and sample with high lateral and spectral resolution.^[^ ^30^ ^]^ In an inelastic electron tunneling spectroscopy (IETS), tunneling electrons sense spin‐flip excitations of a magnetic impurity coupled to the itinerant electrons of a metal,^[^ ^31^, ^32^ ^]^ which manifest themselves in differential conductance (d*I*/d*V*) spectra as pairs of steps symmetric to the Fermi level *E*_F_. This can be rationalized by a phenomenological spin Hamiltonian^[^ ^33^, ^34^ ^]^:

(1) $$H=D{\hat{S}}_{z}^{2}+E\left({\hat{S}}_{x}^{2}-{\hat{S}}_{y}^{2}\right),$$

where *D* and *E* are the axial and transverse anisotropy parameters and ${\hat{S}}_{x,y,z}$ are the three components of the spin operator along the corresponding axes. When magnetic moments are in close vicinity, spins can interact via orbital overlapping^[^ ^35^, ^36^ ^]^, through RKKY interactions,^[^ ^37^ ^]^ or superexchange interaction, which can lead to collective spin eigenstates and additional spin‐flip excitations at higher energies.

Here, we compare the synthesis and spin excitations of a prototypical iron based metal‐organic spin chain on Ag(111) and superconducting Pb(111) using PTO molecules as ligands.^[^ ^38^ ^]^ Using STM and AFM, we have found that the metal‐organic structures are identical on both substrates. STS measured at 1 K on Fe sites reveals a series of low‐energy steps attributed to spin excitations of the iron magnetic moment. On Ag(111), we observe steps at ±20 meV but not on Pb(111), which are attributed to long range spin‐spin coupling between the irons of the metal‐organic chain.

## 2 Results and Discussions

**Figure 1:**
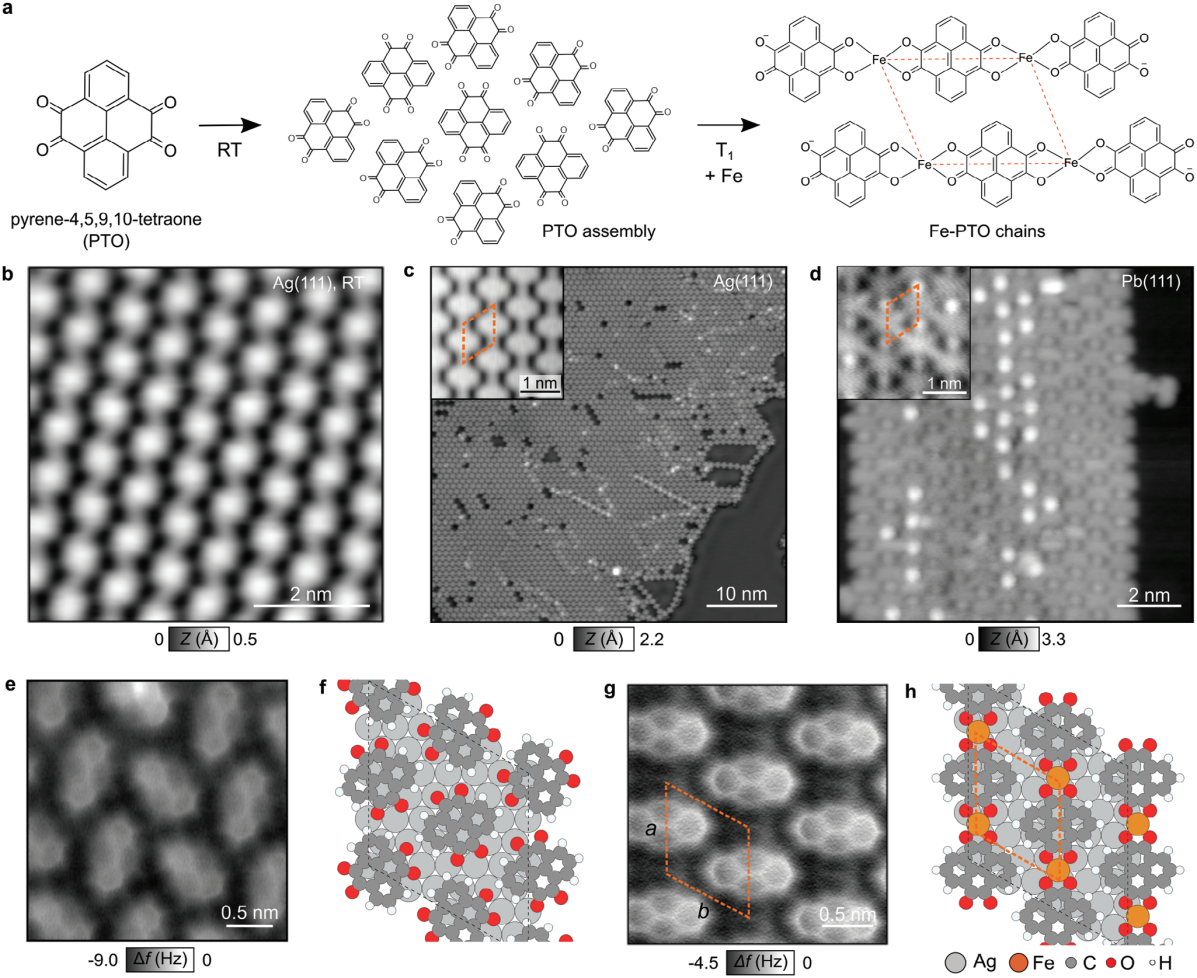
Hierarchical synthesis of the Fe‐PTO spin chain. a) Scheme of the reaction pathway using pyrene‐4,5,9,10‐tetraone precursor. Molecules self‐assemble into a supramolecular network stabilized by hydrogen bonds. Annealing the substrate to *T*_1_ ≈ 120–150 °C while depositing Fe adatoms leads to the formation of the Fe‐PTO chains. b) STM image of the PTO supramolecular network on Ag(111), (*I*_t_ = 1 pA, *V*_s_ = 500 mV). c) STM image of the Fe‐PTO chains on Ag(111) (*I*_t_ = 1 pA, *V*_s_ = 200 mV). The inset shows a close‐up STM image of the structure. d) STM image of the Fe‐PTO structure on Pb(111),(*I*_t_ = 80 pA, *V*_s_ = 600 mV, inset: *I*_t_ = 100 pA, *V*_s_ = 50 mV). e) AFM image with a CO‐terminated tip of the PTO assembly on Ag(111), (*f*_0_= 26.2 kHz, *A* = 50 pm). f) Relaxed structure calculated by DFT of the PTO assembly in registry with the Ag(111). g) AFM image of the Fe‐PTO chains. The orange dashed line shows the Fe–Fe arrangement in the metal–organic network with lattice parameters *a* = 8.7 Å and *b* = 9.0 Å. h) Relaxed structure of Fe‐PTO chain on Ag(111) obtained by DFT calculations.

The reaction pathway of Fe adatoms and PTO precursors into spin chains is shown in **Figure** **1**a. Experimentally, PTO molecules are sublimed onto the substrate kept at room temperature, leading to extended 2D PTO assemblies stabilized by hydrogen bonds (Figure 1b, see Methods). In a second step, Fe adatoms are deposited while annealing the substrate hosting the supramolecular assemblies to *T*_1_ = 120–150°C. This induces the PTO‐Fe coordination and the formation of the spin chain (Figure 1c,d). The oxidative potential of the bridging ligand stabilizes the Fe(II) dication.^[^ ^39^ ^]^

Using AFM imaging with CO‐terminated tips,^[^ ^40^ ^]^ we elucidate the chemical structure of the PTO assembly and the Fe‐PTO spin‐chains on Ag(111) (Figure 1e,g). PTO molecules first self‐assemble into an hexagonal arrangement of molecules rotated by 100° with respect to each other, the structure of which is confirmed by density functional theory (DFT) calculations (Figure 1f). The supramolecular network, in registry with the Ag(111) lattice, is stabilized by intermolecular hydrogen bonds between neighboring ketone side groups and peripheral hydrogen atoms (C═ O⋅⋅⋅H─C).^[^ ^41^ ^]^

**Figure 2:**
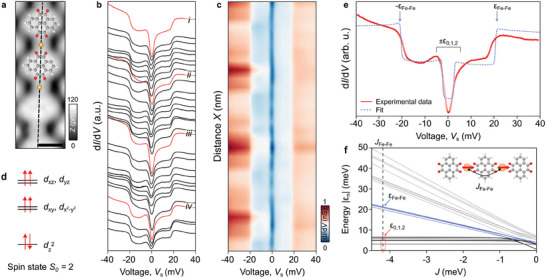
Spin excitations of the Fe‐PTO chain on Ag(111). a) STM image of a PTO‐Fe chain (*I*_t_ = 100 pA, *V*_s_ = 50 mV). Scale bar: 500 pm. b) Series of d*I*/d*V* spectra recorded along the Fe‐PTO chain of a). Spectra measured around Fe sites are colored in red (*I*_t_ = 300 pA, *V*_s_ = 35 mV, *A*_mod_ = 1 mV). c) d*I*/d*V*(V_s_,X) cross‐section of the datatset shown in b. d) Spin ground state of Fe calculated by DFT. It shows a spin ground state *S* = 2 and the 3*d* energy levels. e) Experimental d*I*/d*V* spectrum of Fe (red curve) and the simulated curve (blue dotted line) considering two ferromagnetically coupled Fe spins (see inset of f)) (*I*_t_ = 1.5 nA, *V*_s_ = 35 mV). The simulated curve shows a dip around *E*_F_ denoted ±ε_0,1,2_ and a pair of steps at ±20 meV (±ε_Fe–Fe_) marked by a blue arrow. f) Spin excitation energies as a function of the spin‐spin exchange energy *J*. The magnetic exchange energy corresponding to the Fe‐PTO simulated curve is shown with a dashed line along *J* = *J*_Fe‐Fe_).

In Figure 1g, the Fe coordination to oxygen atoms along the Fe‐PTO chains is unambiguously resolved. The bonding geometry indicates a coordination number of four for each Fe atom as it is bound to four oxygen atoms from two neighboring PTO molecules. For completeness, we also compared the relaxed structures by DFT of Ag‐ and Fe‐coordinated PTO networks and found that the Fe‐coordinated system is 5.40 eV more energetically favorable than its Ag counterpart. The Fe‐PTO molecular network is found in registry with the Ag surface with lattice parameters *a* = 8.7 Å and *b* = 9.0 Å as shown with orange dashed lines in Figure 1g. DFT calculations further indicate a *S* = 2 high‐spin state of the Fe^2 +^ complex (**Figure** **2**d; Figure S1b, Supporting Information) resulting from the coordination with the PTO molecules^[^ ^13^, ^16^ ^]^ We also acquired constant‐height d*I*/d*V* maps at e*V*_s_ ±1.8 meV and ±20 meV (Figure S6c, Supporting Information). At both energies, a homogeneous DOS distribution is observed at PTO molecules with a slightly darker contrast at Fe sites which reflects the spin excitation dips of the corresponding d*I*/d*V* spectra.

To experimentally confirm the Fe spin state, we performed a series of d*I*/d*V* spectra taken along a Fe‐PTO chain (dashed line of Figure 2a). At Fe sites, single‐point d*I*/d*V* spectra colored in red in Figure 2b systematically show a pronounced dip around *E*_F_ marked ±ε_0,1,2_ accompanied by one pair of symmetric steps at about ±20 meV (±ε_Fe‐Fe_). The dip is assigned to low‐energy spin‐flip excitations made possible by the MA of the Fe atom in contact with the Ag(111) substrate, while steps at ±20 meV are attributed to collective excitations between neighboring Fe spins. The d*I*/d*V* lineshape is consistently reproduced on molecule sites, which suggests the delocalization of these spin‐flip processes across the entire Fe‐PTO chains. We assume that, instead of a direct overlap of Fe *d*‐orbitals, a long‐range magnetic coupling between Fe atoms is the consequence of such spin‐spin coupling.

A representative d*I*/d*V* spectrum acquired at Fe sites of a single chain or in the 2D assembly of chains is shown in red in Figure 2e. To rationalize this, we first simulated the spectrum using the perturbative model developed by Ternes (see Methods) considering a single magnetic impurities of spin state *S* = 2. The best agreement between simulation and experiment is only obtained for the low‐energy spin excitations (i.e., |*eV*_s_| = ε_0,1,2_ ⩽ 10 meV) using the parameters *g* = 2.1, *D* = –0.8 meV and *E* = 0.25 meV, while taking into account the substrate electron bath (i.e., potential scattering of *U* = 0.2 meV and Kondo scattering of *J*_*K*_ρ_*s*_ = –0.09 meV). As both axial and transverse anisotropy are considered in simulations, five spin states can be constructed using the single Fe impurity model. Considering the selection rule, only excitations with Δ*m*_*z*_ = ±1 or 0 are possible. The ground state Ψ_0_ has the largest weight in |*m*_*z*_〉 = |±2〉 allowing the transition to Ψ_1_ (Δ*m*_*z*_ = 0), Ψ_2_ and Ψ_3_ (Δ*m*_*z*_ = ±1). The resulting low‐energy spin excitations from simulation are then ε_0_ = ±0.2 meV, ε_1_ = ±1.9 meV, and ε_2_ = ±3.3 meV, respectively. In Figure 2e, we marked the region of these spin excitations energies ε_0,1,2_ which are responsible for the dip of the experimental spectra. Note also that to distinguish these low excitation energies of single atoms usually requires the use of a decoupling layer to reduce the DOS contribution from the normal metal substrate.^[^ ^32^, ^42^, ^43^ ^]^ In our system, we assume that such decoupling from the Ag(111) is favored by the coordination with surrounding PTO molecules.^[^ ^13^, ^16^ ^]^

With the exception of the ε_0,1,2_ spin excitations, the simulation using a single impurity model is unable to reproduce the ε_Fe‐Fe_ steps at ±20 meV (blue arrow of Figure 2e). Inspired by spin‐ladder compounds^[^ ^44^ ^]^ and other 2D MOFs on surfaces,^[^ ^16^ ^]^ we next considered a chain of coupled spins with magnetic exchange interaction *J* as depicted in the inset of Figure 2f. The spin Hamiltonian now writes as:

(2) $$H=\sum_{i}^{2}H_{i}+J\left(\vec{S_{1}}\cdot \vec{S_{2}}\right),$$

where $H_{i}$ is the Hamiltonian of a single Fe. *J*_Fe‐Fe_ corresponds to the magnetic exchange coupling between the Fe spins along the Fe‐PTO chain.

According to DFT calculations of the Fe‐PTO chain at fixed geometry in a collinear configuration (see Methods), an antiferromagnetic (AfM) coupling is favored as compared to a ferromagnetic (FM) one by about 2 meV per Fe atom. This energetic preference is only 0.73 meV per Fe atom for the relaxed structures on Ag (Figure S2, Supporting Information). Note also that surface‐induced MA and spin‐orbit coupling are not considered in the spin‐polarized DFT calculations, which makes the assignment of magnetic ground state in the system challenging.

We next simulate using the Ternes code^[^ ^30^ ^]^ d*I*/d*V* spectra considering both AfM and FM order between *S* = 2 spins with a linear Heisenberg exchange coupling of *J* = ±4.1 meV and fit parameters *g* = 2.11, *D* = ‐0.8 meV, *E* = 0.21 meV, *U* = 0.2, *J*ρ_s_ = −0.09, *T*_eff_ = 1.2 K (SI Figure S3). The best agreement with the experimental data is only obtained using a FM coupling (blue dotted line of Figure 2e; see also Figure S3 and Figure S7, Supporting Information), which feature the low‐energy spin excitations from Fe together with the kinks at ±20 meV. For Fe spins at the end of the chain, we still observe the kinks together with a sharper dip at zero‐energy (Figure S7, Supporting Information), that we ascribe to a reduction of the iron spin state as compared to that in the chain middle (see Figure S7f, Supporting Information).^[^ ^45^ ^]^

Figure 2f last shows the evolution of these spin excitation energies as a function of *J*. The low‐energy spin excitations ε_0,1,2_ arise from the MA of the Fe atom adsorbed on Ag(111) and remain constant and below 10 meV as *J* increases. They are responsible for the dip centered to *E*_F_ observed in the d*I*/d*V* spectra. In addition, a series of spin excitations are observed at higher energy (⩾ 10 meV), which linearly increases with *J*. From this plot, we estimate the magnetic exchange interaction *J*_Fe–Fe_ = –4.1 meV to be ferromagnetic (marked by a dashed line in Figure 2f) as it best reproduces the ε_0,1,2_ dip and the steps ε_Fe‐Fe_ at |e*V*_s_| = 20 meV (see also Figure S3, Supporting Information, for the AfM case). However, only spin‐sensitive experimental techniques such as X‐ray Magnetic Circular Dichroism (XMCD)^[^ ^5^ ^]^ or spin‐polarized STM (SP‐STM)^[^ ^46^ ^]^ can unambiguously address the magnetic order of the Fe‐PTO chains on Ag(111). Future experiments will attempt to address this in more details.

**Figure 3:**
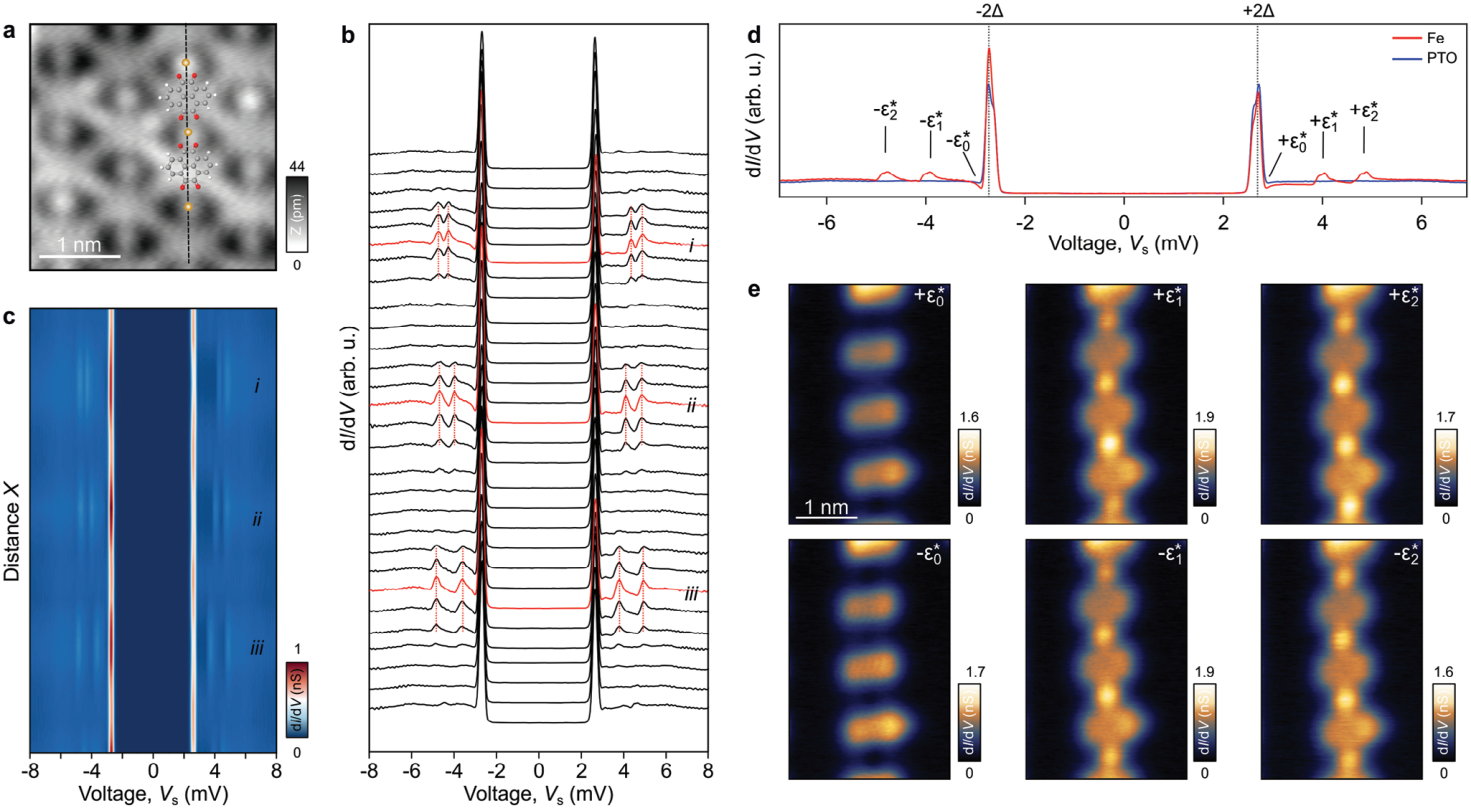
Spin excitations of the Fe‐PTO chain on Pb(111). a) STM image of the Fe‐PTO chain on Pb(111), (*I*_t_ = 80 pA, *V*_s_ = 9 mV). b) Series of d*I*/d*V* spectra recorded along the black line of (a). d*I*/d*V* spectra corresponding to Fe sites are plotted in red (*I*_t_ = 300 pA, *V* = 8 mV, *A*_mod_ = 50 µV). c) d*I*/d*V* cross‐section showing asymmetric coherence peaks and resonances outside the gap attributed to spin excitations of the Fe sites marked *i*, *ii* and *iii*, respectively. These features are absent on PTO molecules. d) Representative d*I*/d*V* spectra at Fe site (red) as compared to PTO molecule (blue), (*I*_t_ = 800 pA, *V*_s_ = 7 mV, *A*_mod_ = 18 µV). e) d*I*/d*V* maps recorded at the spin‐excitation energy of $\pm \varepsilon_{0}^{*}$, $\pm \varepsilon_{1}^{*}$ and $\pm \varepsilon_{2}^{*}$, respectively).

Using the same preparation procedure, we next synthesize the Fe‐PTO chains on Pb(111). The STM image **Figure** **3**a shows the lattice with parameters *a* = 8.8 Å and *b* = 9.1 Å in agreement with the structure on Ag(111), where iron atoms are observed as spots. Note also that, in Figure 1d, a few of these spots appear much brighter and correspond to Pb atoms coordinated between two PTO molecules (i.e., Pb^2 +^). Since Pb(II) is diamagnetic, they do not show any spin excitations as evidenced in the d*I*/d*V* spectra of Figure S11b (Supporting Information). Figure 3c shows the d*I*/d*V* cross‐section along a Fe‐PTO chain of a molecular domain (dashed line of Figure 3a) using a superconducting Pb‐tip at 1 K. Figure 3b is a waterfall plot of the same dataset. *i*, *ii* and *iii* refers to the position of Fe atoms in both Figure 3b,c. In d*I*/d*V* spectra on bare Pb(111), the depletion of the density of states around *E*_F_ due to the superconducting state of the surface (Δ_sample_) and the tip (Δ_tip_) is observed as a gap separated by two sharp coherence peaks located at 2Δ = ±2.7 meV.

On PTO molecules, the position and lineshape of the superconducting gap Δ are identical to the bare Pb(111) (blue curve of Figure 3d). This indicates that the superconducting state is unaffected by the presence of the PTO precursor implying the absence of any magnetic interaction from the molecule. At Fe sites (red curve in Figure 3b), d*I*/d*V* spectra show an asymmetric superconducting gap framed by two pairs of resonances at $\pm \varepsilon_{1}^{*}$ = ±3.9 meV and $\pm \varepsilon_{2}^{*}$ = ±4.7 meV outside the gap. In analogy to the Fe‐PTO chain on Ag(111), we assigned these features to the series of low‐energy spin excitations of the Fe(II)‐PTO complex on Pb(111). Comparing spectra at the *i*, *ii* and *iii* locations further shows slight variations in energy of these resonances (Figure S7 and Table S1, Supporting Information), which we attribute to a variation of the coupling of Fe spins with the Pb substrate.^[^ ^14^, ^47^ ^]^

Coordinated Fe adatoms on Pb(111) are in a *S* = 2 high spin state. On superconductor, the energies of spin excitations in d*I*/d*V* spectra are symmetric with respect to *E*_F_ but shifted by ±2Δ = ±2.7 meV due to the superconducting state of the tip and the substrate.^[^ ^48^, ^49^ ^]^ Experimental resonances $\pm \varepsilon_{1}^{*}$ and $\pm \varepsilon_{2}^{*}$ on Pb(111) thus correspond to spin excitation energies |ε_1_| = $|\varepsilon_{1}^{*}|$ − 2Δ = 1.2 meV and |ε_2_| = $|\varepsilon_{2}^{*}|$ − 2Δ = 2.0 meV, respectively. They do not match the ones observed on Ag(111) as shown in the **Table** **1**.

**Table 1:** Comparison of (*D*, *E*) parameters and spin‐excitation energies |ε_*n*_| in meV of the Fe‐PTO chain adsorbed on Ag(111) and on Pb(111) extracted from simulations.

	on Ag(111)	on Pb(111)
(*D*, *E*)	(‐0.8, 0.25)	(‐0.51, 0.1)
|ε_0_|	0.20	0.12
|ε_1_|	1.90	1.24
|ε_2_|	3.30	2.07
|ε_Fe‐Fe_|	20	—

To explain this discrepancy, we next reproduce d*I*/d*V* spectra for a single Fe(II) impurity on a metal surface using the phenomenological spin hamiltonian with the parameters *D* = –0.51 meV and *E* = 0.1 meV, *U* = 0.01 meV *J*_*K*_ρ_*s*_ = –0.27 meV (Figure S9a, Supporting Information). This spectrum was then shifted by ±2Δ at each side of the Fermi level as schematically shown in Figure S9b (Supporting Information), which now provides a good agreement with the experimental data of Figure 3d. The excitation energies obtained from simulation (see Table 1) are ε_0_ = 0.12 meV, ε_1_ = 1.24 meV, and ε_2_ = 2.07 meV, respectively. The ε_0_ energy on Pb(111) should emerge at $\varepsilon_{0}^{*}$ = ±2.82 meV as a conductance dip in the spectra (see Figure S9, Supporting Information). However, the energy positions of $\varepsilon_{0}^{*}$ are close to the superconducting coherence peaks, whose high conductance hinders their observation as clearly as the $\varepsilon_{1}^{*}$ and $\varepsilon_{2}^{*}$ resonance peaks. In Figure S9d,e (Supporting Information), we show d*I*/d*V* zooms of the coherence peaks allowing to attribute the ε_0_ spin excitations to the small conductance dips denoted $\varepsilon_{0}^{*}$.

Figure 3e shows d*I*/d*V* maps acquired at energies $\pm \varepsilon_{0}^{*}$, $\pm \varepsilon_{1}^{*}$ and $\pm \varepsilon_{2}^{*}$, respectively. For the $\varepsilon_{1}^{*}$ and $\varepsilon_{2}^{*}$, the DOS is more pronounced at Fe sites while PTO molecules show a homogeneous background, which indicates the localization of the spin‐flip process at the Fe atoms. Note also that small variation in intensity at Fe atoms can be observed, which is due to the slight shift in energy of these spin excitations as a function of the position (Figure 3b). The map at $\pm \varepsilon_{0}^{*}$ mainly shows the distribution of DOS at PTO molecules with a darker contrast at Fe sites, thus reflecting the dips of d*I*/d*V* spectra recorded at Fe atoms (Figure 3d).

We last applied a magnetic field of 0.5 T perpendicular to the sample in order to quench the superconducting state of both tip and substrate (Figure S12, Supporting Information). By inducing such a metallic state, the spin excitation energies ε_*n*_ of Fe are not shifted anymore by the tip/sample superconducting gaps and now appears near *E*_F_. Similar to Ag(111), the experimental d*I*/d*V* spectrum thus shows a dip centered at *E*_F_ (red spectra, Figure S12c, Supporting Information) which is not observed on bare Pb(111) (blue spectra, Figure S12c, Supporting Information).

## 3 Conclusion

In contrast to Ag(111), spin‐spin interactions between neighboring Fe atoms expected at large energy were never observed on Pb(111) with and without an external magnetic field (Figure S12, Supporting Information). As reported in Reference,^[^ ^14^ ^]^ this difference between the two substrates is likely governed by their band structures. The Ag(111) surface exhibits a Shockley surface state at about –68 meV with a parabolic dispersion close to the Γ‐point,^[^ ^50^ ^]^ while Pb(111) has a flat dispersion around *M*‐ and *K*‐point at –2.2 eV.^[^ ^51^ ^]^ We think that the superexchange interaction between Fe atoms depends on the amount of charge transfer from the substrate to the Fe‐PTO system. The strong hybridization of the molecule with the Ag(111) states might vary the charge distribution of Fe(II)‐PTO bonding and the strength of the superexchange mechanism (Figures S2, Supporting Information).

In conclusion, we used coordination chemistry to synthesize an iron‐based metal‐organic ladder using PTO precursors as ligands. We compared the magnetic signature of the high‐spin Fe(II) using tunneling spectroscopy at low temperature. The iron coordination with PTO decouples the Fe(II) magnetic moment from both substrate,^[^ ^14^, ^47^ ^]^ allowing the signature of spin excitations in d*I*/d*V* spectra. The MA induced by both surfaces leads to low‐energy steps related to spin excitations of the iron *S* = 2 spins. On the Ag(111) surface these occurrences are accompanied with steps at larger voltages, which we ascribe to the signature of a spin‐spin coupling between neighboring Fe atoms due to superexchange interaction through the PTO ligands. On Pb(111), these steps are not experimentally observed which could be explained by the depletion of electronic states on Pb(111) as compared to Ag(111). This likely decreases the hybridization of the PTO precursors with the surface and the amount of charge transfer to the Fe‐PTO bonding.

Overall, our results demonstrate the synthesis of a two‐dimensional metal‐organic structure on a superconductor that will motivate the design and fabrication of low‐dimensional magnetic superconductor hybrids with potentially protected spin states.^[^ ^22^, ^23^ ^]^

## Conflict of Interest

The authors declare no conflict of interest.

## Supporting information

Supporting Information

## Data Availability

The data that support the findings of this study are available from the corresponding author upon reasonable request.

## References

[1] X. Zhang , Y. Zhou , B. Cui , M. Zhao , F. Liu , Nano Lett. 2017, 17, 6166. 28898086 10.1021/acs.nanolett.7b02795

[2] T. Takenaka , K. Ishihara , M. Roppongi , Y. Miao , Y. Mizukami , T. Makita , J. Tsurumi , S. Watanabe , J. Takeya , M. Yamashita , K. Torizuka , Y. Uwatoko , T. Sasaki , X. Huang , W. Xu , D. Zhu , N. Su , J.‐G. Cheng , T. Shibauchi , K. Hashimoto , Sci. Adv. 2021, 7, eabf3996. 33731356 10.1126/sciadv.abf3996 PMC7968839

[3] W. Li , L. Sun , J. Qi , P. Jarillo‐Herrero , M. Dincă , J. Li , Chem. Sci. 2017, 8, 2859. 28553524 10.1039/c6sc05080h PMC5428024

[4] J. G. Park , B. A. Collins , L. E. Darago , T. Runčevski , M. E. Ziebel , M. L. Aubrey , H. Z. H. Jiang , E. Velasquez , M. A. Green , J. D. Goodpaster , J. R. Long , Nat. Chem. 2021, 13, 594. 33859391 10.1038/s41557-021-00666-6

[5] J. Lobo‐Checa , L. Hernández‐López , M. M. Otrokov , I. Piquero‐Zulaica , A. E. Candia , P. Gargiani , D. Serrate , F. Delgado , M. Valvidares , J. Cerdá , A. Arnau , F. Bartolomé , Nat. Commun. 2024, 15, 1858. 38424075 10.1038/s41467-024-46115-z PMC10904770

[6] J. Pitcairn , M. A. Ongkiko , A. Iliceto , P. J. Speakman , S. Calder , M. J. Cochran , J. A. M. Paddison , C. Liu , S. P. Argent , A. J. Morris , M. J. Cliffe , J. Am. Chem. Soc. 2024, 146, 19146. 38953583 10.1021/jacs.4c04102 PMC11258693

[7] M. G. Yamada , H. Fujita , M. Oshikawa , Phys. Rev. Lett. 2017, 119, 057202. 28949730 10.1103/PhysRevLett.119.057202

[8] Y. Misumi , A. Yamaguchi , Z. Zhang , T. Matsushita , N. Wada , M. Tsuchiizu , K. Awaga , J. Am. Chem. Soc. 2020, 142, 16513. 32623880 10.1021/jacs.0c05472

[9] Z. F. Wang , Z. Liu , F. Liu , Nat. Comm. 2013, 4, 1471. 10.1038/ncomms2451 23403572

[10] S. Stepanow , N. Lin , J. V. Barth , J. Phys.: Condens. Matter 2008, 20, 184002.

[11] C. Carbone , S. Gardonio , P. Moras , S. Lounis , M. Heide , G. Bihlmayer , N. Atodiresei , P. H. Dederichs , S. Blügel , S. Vlaic , A. Lehnert , S. Ouazi , S. Rusponi , H. Brune , J. Honolka , A. Enders , K. Kern , S. Stepanow , C. Krull , T. Balashov , A. Mugarza , P. Gambardella , Adv. Funct. Mater. 2011, 21, 1212.

[12] L. Dong , Z. Gao , N. Lin , Prog. Surf. Sci. 2016, 91, 101.

[13] P. Gambardella , S. Stepanow , A. Dmitriev , J. Honolka , F. M. F. de Groot , M. Lingenfelder , S. S. Gupta , D. D. Sarma , P. Bencok , S. Stanescu , S. Clair , S. Pons , N. Lin , A. P. Seitsonen , H. Brune , J. V. Barth , K. Kern , Nature Mater. 2009, 8, 189. 19182787 10.1038/nmat2376

[14] N. Abdurakhmanova , T.‐C. Tseng , A. Langner , C. S. Kley , V. Sessi , S. Stepanow , K. Kern , Phys. Rev. Lett. 2013, 110, 027202. 23383936 10.1103/PhysRevLett.110.027202

[15] M.‐M. Xu , Q. Chen , L.‐H. Xie , J.‐R. Li , Coord. Chem. Rev. 2020, 421, 213421.

[16] T. R. Umbach , M. Bernien , C. F. Hermanns , A. Krüger , V. Sessi , I. Fernandez‐Torrente , P. Stoll , J. I. Pascual , K. J. Franke , W. Kuch , Phys. Rev. Lett. 2012, 109, 267207. 23368613 10.1103/PhysRevLett.109.267207

[17] M. A. Ruderman , C. Kittel , Phys. Rev. 1954, 96, 99.

[18] T. Kasuya , Prog. Theor. Phys. 1956, 16, 45.

[19] K. Yosida , Phys. Rev. 1957, 106, 893.

[20] B. Mallada , P. Błoński , R. Langer , P. Jelínek , M. Otyepka , B. de la Torre , ACS Appl. Mater. Interfaces 2021, 13, 32393. 34227386 10.1021/acsami.1c04693

[21] C. Li , R. Robles , N. Lorente , S. K. Mahatha , S. Rohlf , K. Rossnagel , A. Barla , B. V. Sorokin , S. Rusponi , P. Ohresser , S. Realista , P. N. Martinho , T. Jasper‐Toennies , A. Weismann , R. Berndt , M. Gruber , ACS Nano 2023, 17, 10608. 37224165 10.1021/acsnano.3c01595 PMC10278185

[22] B. W. Heinrich , L. Braun , J. I. Pascual , K. J. Franke , Nature Phys. 2013, 9, 765.

[23] K. Vaxevani , J. Li , S. Trivini , J. Ortuzar , D. Longo , D. Wang , J. I. Pascual , Nano Lett. 2022, 22, 6075. 35895892 10.1021/acs.nanolett.2c00924

[24] E. Liebhaber , L. M. Rütten , G. Reecht , J. F. Steiner , S. Rohlf , K. Rossnagel , F. von Oppen , K. J. Franke , Nat. Commun. 2022, 13, 2160. 35443753 10.1038/s41467-022-29879-0 PMC9021194

[25] K. Pöyhönen , A. Westström , J. Röntynen , T. Ojanen , Phys. Rev. B 2014, 89, 115109.

[26] M. O. Soldini , F. Küster , G. Wagner , S. Das , A. Aldarawsheh , R. Thomale , S. Lounis , S. S. P. Parkin , P. Sessi , T. Neupert , Nature Phys. 2023, 19, 1745.

[27] B. Jäck , Y. Xie , A. Yazdani , Nat. Rev. Phys. 2021, 3, 541.

[28] G. Ahmadi , K. J. Franke , Appl. Surf. Sci. 2016, 373, 2.

[29] L. Yan , O. J. Silveira , B. Alldritt , S. Kezilebieke , A. S. Foster , P. Liljeroth , ACS Nano 2021, 15, 17813. 34730941 10.1021/acsnano.1c05986 PMC8613900

[30] M. Ternes , New J. Phys. 2015, 17, 063016.

[31] A. J. Heinrich , J. A. Gupta , C. P. Lutz , D. M. Eigler , Science 2004, 306, 466. 15358866 10.1126/science.1101077

[32] C. F. Hirjibehedin , C.‐Y. Lin , A. F. Otte , M. Ternes , C. P. Lutz , B. A. Jones , A. J. Heinrich , Science 2007, 317, 1199. 17761877 10.1126/science.1146110

[33] D. Gatteschi , R. Sessoli , J. Villain , *Molecular Nanomagnets*, Oxford University Press, 2006.

[34] D. Jacob , Phys. Rev. B 2018, 97, 075428.

[35] C. F. Hirjibehedin , C. P. Lutz , A. J. Heinrich , Science 2006, 312, 1021. 16574821 10.1126/science.1125398

[36] D.‐J. Choi , R. Robles , J.‐P. Gauyacq , M. Ternes , S. Loth , N. Lorente , Phys. Rev. B 2016, 94, 085406. 10.1021/acs.nanolett.7b02882 28872317

[37] J. Girovsky , J. Nowakowski , M. E. Ali , M. Baljozovic , H. R. Rossmann , T. Nijs , E. A. Aeby , S. Nowakowska , D. Siewert , G. Srivastava, C. Wäckerlin , J. Dreiser , S. Decurtins , S.‐X. Liu , P. M. Oppeneer , T. A. Jung , N. Ballav , Nat. Commun. 2017, 8, 15388. 28530247 10.1038/ncomms15388 PMC5458152

[38] A. D. Pía , M. Riello , J. Lawrence , D. Stassen , T. S. Jones , D. Bonifazi , A. De Vita , G. Costantini , Chem‐Eur J 2016, 22, 8105. 27071489 10.1002/chem.201600368 PMC5074249

[39] D. Skomski , C. D. Tempas , G. S. Bukowski , K. A. Smith , S. L. Tait , J. Chem. Phys. 2015, 142, 101913. 25770502 10.1063/1.4906894

[40] L. Gross , N. Moll , F. Mohn , A. Curioni , G. Meyer , F. Hanke , M. Persson , Phys. Rev. Lett. 2011, 107, 086101. 21929180 10.1103/PhysRevLett.107.086101

[41] R. Pawlak , S. Clair , V. Oison , M. Abel , O. Ourdjini , N. A. A. Zwaneveld , D. Gigmes , D. Bertin , L. Nony , L. Porte , ChemPhysChem 2009, 10, 1032. 19334020 10.1002/cphc.200900055

[42] A. F. Otte , M. Ternes , K. von Bergmann , S. Loth , H. Brune , C. P. Lutz , C. F. Hirjibehedin , A. J. Heinrich , Nature Phys. 2008, 4, 847.

[43] T. Choi , C. D. Ruggiero , J. A. Gupta , J. Vac. Sci. Technol. B 2009, 27, 887.

[44] R. A. L. Silva , M. Almeida , J. Mater. Chem. C 2021, 9, 10573.

[45] X. Meng , J. Möller , M. Mansouri , D. Sánchez‐Portal , A. Garcia‐Lekue , A. Weismann , C. Li , R. Herges , R. Berndt , ACS Nano 2023, 17, 1268. 36440841 10.1021/acsnano.2c09310 PMC10711789

[46] R. Wiesendanger , Rev. Mod. Phys. 2009, 81, 1495.

[47] K. J. Franke , G. Schulze , J. I. Pascual , Science 2011, 332, 940. 21596987 10.1126/science.1202204

[48] P. Berggren , J. Fransson , Phys. Rev. B 2015, 91, 205438.

[49] P. Berggren , J. Fransson , Europhys. Lett. 2015, 108, 67009.

[50] L. Bürgi , L. Petersen , H. Brune , K. Kern , Surf. Sci. 2000, 447, L157.

[51] K. Würde , A. Mazur , J. Pollmann , Phys. Rev. B 1994, 49, 7679. 10.1103/physrevb.49.7679 10009513

