# Supplementary material for: Spin Excitations of High Spin Iron(II) in Metal–Organic Chains on Metal and Superconductor
Source: Adv Sci (Weinh). 2024 Dec 24;12(7):2412351. doi: 10.1002/advs.202412351 (PMC11831492; doi:10.1002/advs.202412351)
Supplement: Supplementary file 1 — Supporting Information [file ADVS-12-2412351-s001.pdf]

ADVANCED SCIENCE
Open Access

Supporting Information

for *Adv. Sci.*, DOI 10.1002/advs.202412351

Spin Excitations of High Spin Iron(II) in Metal–Organic Chains on Metal and Superconductor

Jung-Ching Liu, Chao Li, Outhmane Chahib, Xing Wang, Simon Rothenbühler, Robert Häner, Silvio Decurtins, Ulrich Aschauer, Shi-Xia Liu*, Ernst Meyer and Rémy Pawlak**

Supplementary Information: Spin Excitations of High Spin Iron(II) in Metal-Organic Chains on Metal and Superconductor

Jung-Ching Liu,* Chao Li, Outhmane Chahib, Xing Wang, Simon Rothenbühler, Robert Häner, Silvio Decurtins, Ulrich Aschauer, Shi-Xia Liu,* Ernst Meyer, Rémy Pawlak*

Contents

1. Experimental Methods **S2**
1.1. Sample preparation S2
1.2. STM/AFM measurements S2
1.3. DFT calculations S2

2. Details of DFT calculations **S3**
2.1. Relaxed structure by DFT S3
2.2. Magnetic order and orbital splitting S4

3. Molecular Orbitals of PTO-Fe Chains **S6**

4. Spin excitation spectra on Pb(111) **S8**
4.1. Variation in the spin-flip excitation energies S8
4.2. Tunneling spectra on a Fe cluster S10
4.3. Spin excitations of Fe-PTO/Pb(111) under magnetic field S12

References **S13**

1. Experimental Methods

1.1. Sample preparation

Pyrene-4,5,9,10-tetraone (PTO) can be synthesized in large scale as described by Hu *et al.*[1] Ag(111) and Pb(111) substrates purchased from MaTeck GmbH were cleaned by cycles of Ar^+ sputtering and annealing under ultra-high vacuum (UHV) ($\approx 10^{-10}$ mbar). PTO molecules were then sublimed at 150°C with both substrates remaining at room temperature. The molecule flux was measured by quartz microbalance, which showed 1.6 Å/min at 150°C. Following the molecule deposition, Fe atoms were then evaporated using the e-beam evaporator (EFM3-Focus GmbH) onto surfaces pre-heated to 150°C for Ag(111) and 115°C for Pb(111). Fe atoms were deposited for 1 minute with a measured flux about 10 nA.

1.2. STM/AFM measurements

The structural characterization was carried out under UHV with a low-temperature STM/AFM (4.7 K) purchased from by Omicron GmbH. The microscope is equipped with a qPlus sensor,[2] with eigenfrequency of around 26 kHz and spring constant k of 1800 N.m^{-1}. To enhance the AFM resolution, the tip was functionalized with a CO molecule, which was adsorbed on the cold surface ($\leq$ 15 K) and then gently indent the tip on top of a CO molecule. dI/dV spectra and maps were recorded using the Joule-Thomson STM/AFM at 1 K under UHV. The measurement was performed with the lock-in amplifier with frequency of 613 Hz and modulation amplitude noted in captions using a Low Noise I/V Converter (SP983c) from Basel Precision Instruments. The signal-to-noise ratio of dI/dV spectra was improved by applying a voltage divider (1/100) and a differential amplifier for the bias line. Radio frequency noises were suppressed by avoiding ground loops and using π-filters.

To improve the spectral resolution at 1 K, we used superconducting Pb-tips. A Pb-tip was prepared by cutting a short segment of Pb wire which was then sputtered and annealed in UHV for removing its native oxide. The sharpness and superconducting state of the tip were later improved by indenting into the Pb(111) substrate, until the superconducting gap width shows 4Δ on Pb(111). All fits of the spin excitation spectra were done using the code developed by M. Ternes described here.[3,4]

1.3. DFT calculations

DFT calculations were performed with the Quickstep module[5] of CP2K[6] using the Gaussian and plane waves method (GPW). We used the Perdew-Burke-Ernzerhof (PBE) functional[7] with the Grimme D3 dispersion correction[8] and a Hubbard U correction of 5.0 eV on the Fe 3d states[9,10]. Goedecker, Teter and Hutter (GTH) pseudopotentials[11] were used together with double zeta molecularly optimized basis sets[12]. The orbital transformation (OT) method[13] was used for self-consistent wavefunction optimization.

The Ag(111) surface was modeled as a 4 layer thick slab with the bottom two layers fixed at bulk positions and an approximately 15 Å wide vacuum gap. As a dipole correction[14] did not yield significantly different energies (0.05 eV difference), it was omitted. For adsorption calculations, a hexagonal surface cell with a lattice constant of 20.36 Å was used. For DFT calculations only collinear spins are considered.

2. Details of DFT calculations

2.1. Relaxed structure by DFT

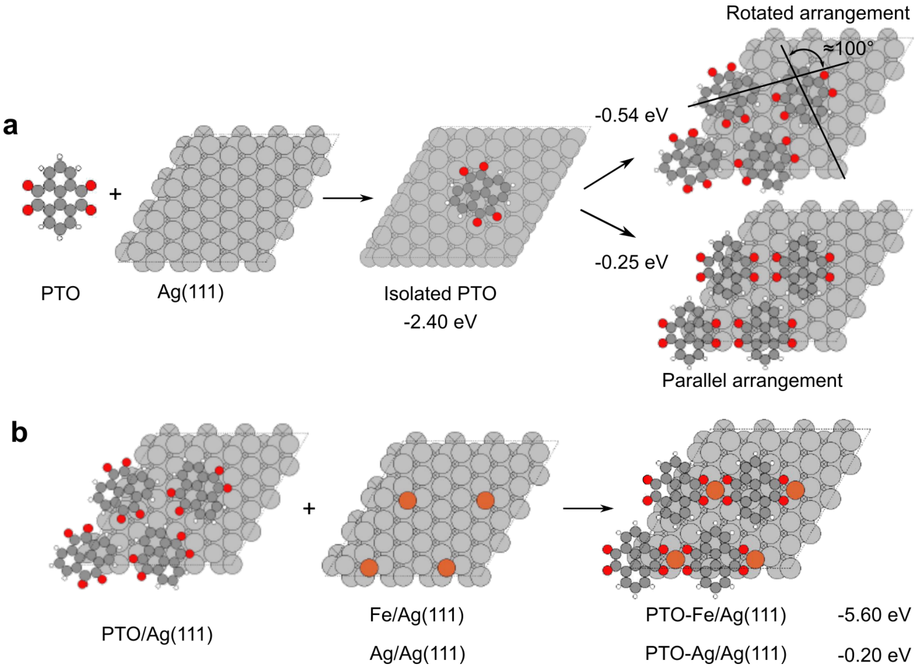

Figure S1. Stability of PTO assemblies and PTO-Fe chains on Ag(111). **a**, For a pure PTO assembly, a rotated arrangement is energetically more favorable by 0.29 eV than the parallel alignment. **b** In presence of Fe, a large energy lowering of 5.60 eV for four PTO and four Fe is associated with the formation of PTO-Fe-PTO chains. This energetic advantage is much smaller (0.20 eV) for Ag adatoms.

To support the observation from STM and AFM, density functional theory (DFT) calculations were performed on Ag(111). The PTO self-assembly can be stabilized through hydrogen bonds among molecules and form either a rotated or parallel arrangement. According to the DFT calculations, a PTO assembly with a rotational angle of about 100° between the axes of adjacent molecules is energetically more favorable than a parallel arrangement (Fig. S1a), which is consistent with the topographic observation as shown in the main text (Figs. 1b and 2a). As shown in Fig. S1b, Fe-adatoms strongly direct the formation of parallel PTO-Fe-PTO chains. This effect is much less marked for Ag adatoms. Nevertheless, the experimental confirmation of Fe-coordination relies on the spectroscopic investigation on magnetic signatures.

2.2. Magnetic order and orbital splitting

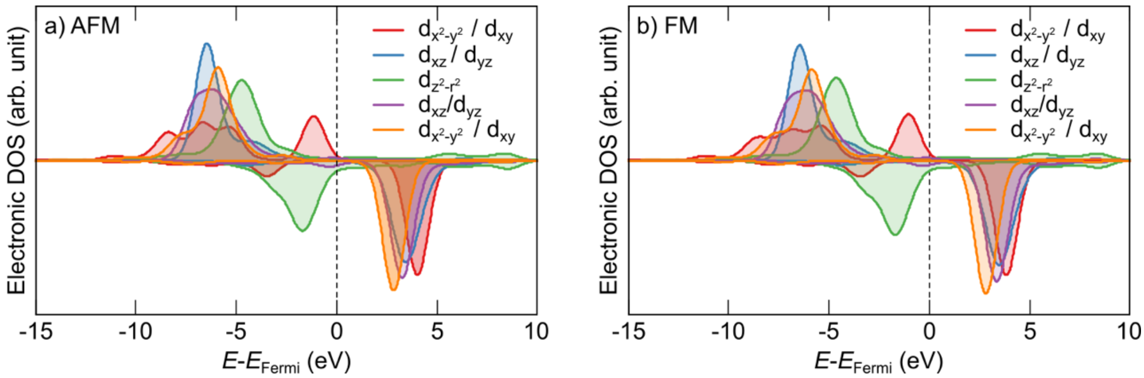

Figure S2. Electronic density of states (DOS) projected onto the $3d$ orbitals of a single (spin up) Fe atom **a**, in the antiferromagnetic and **b**, ferromagnetic spin arrangement.

The magnetic exchange interaction between iron atoms in the Fe-PTO chains has been estimated by DFT calculations (Figure S3). The antiferromagnetic coupling is slightly favored in energy over the ferromagnetic coupling by 0.73 meV per Fe atom. In the ferromagnetic state, the total magnetic moment is 4.00 μ_B per Fe atom while the antiferromagnetic state is 3.88 μ_B per Fe atom. Both of these spin arrangement are in line with the $S = 2$ state. The density of states (DOS) projected on the $3d$ orbitals of a single (spin up) Fe in the antiferromagnetic spin arrangement is shown in Figure S3, which shows that only the $d_{z^2-r^2}$ orbital is doubly occupied. While in the up-spin channel orbitals undergo significant mixing, the orbital splitting can be more clearly seen in the down-spin channel, leading to the orbital diagram shown in Figure 2d of the main text.

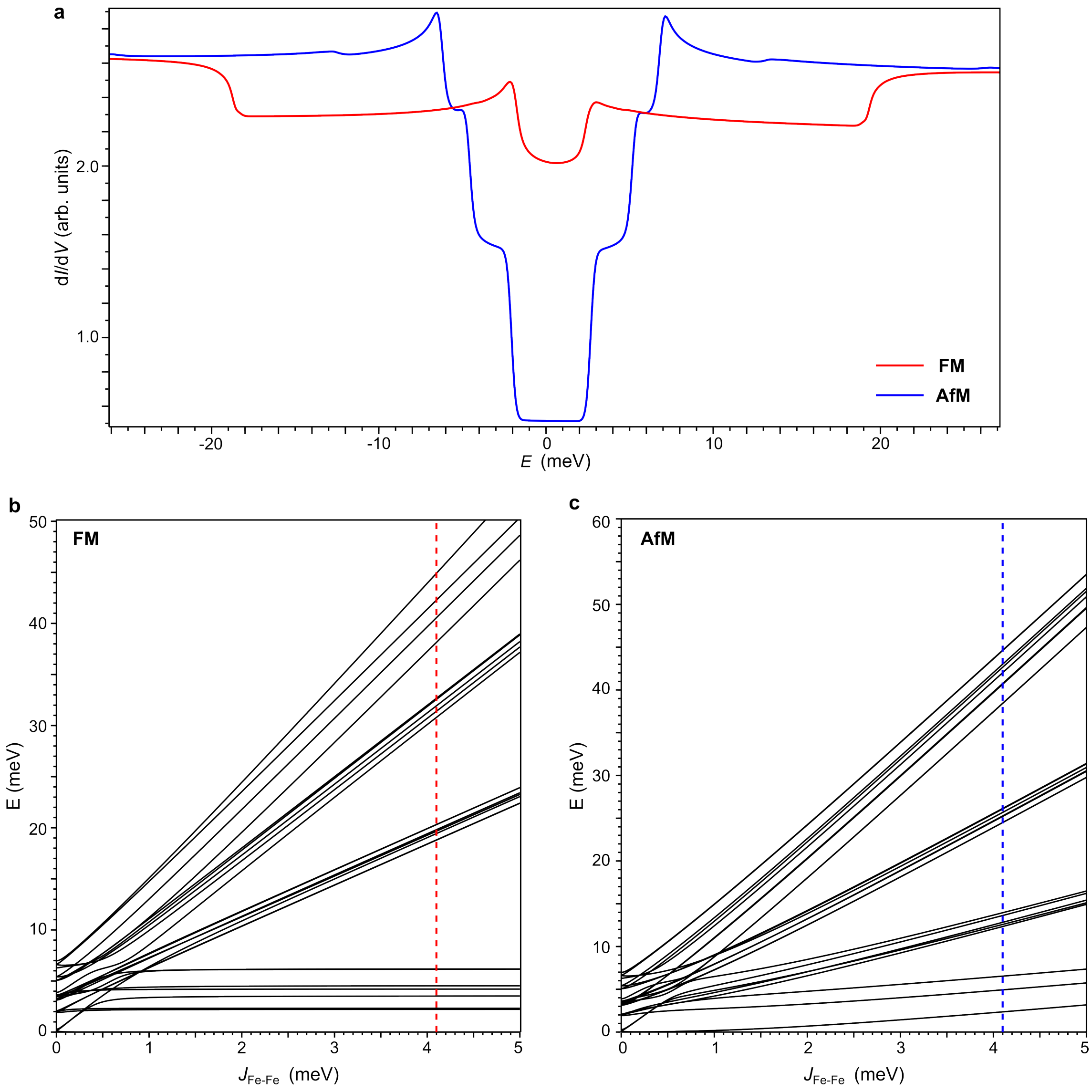

Figure S3. **a,** Simulated dI/dV spectra for S = 2 Fe impurities coupled to a metal with antiferromagnetic (AfM, blue) and ferromagnetic (FM, red) order of $J = J_{\text{Fe-Fe}}$ = 4.1 meV. Parameters for the simulation are g = 2.11, D = -0.8 meV, E = 0.21 meV, U = 0.2, $J\rho_s$ = -0.09, T_{eff} = 1.2 K. **b-c**, Spin excitation energies as a function of the spin-spin exchange energy J for a ferromagnetic (**b**) and antiferromagnetic (**c**) order. The magnetic exchange energy corresponding to the Fe-PTO simulated curve of Figure 2 is shown with a dashed line along $J = J_{\text{Fe-Fe}}$.

3. Molecular Orbitals of PTO-Fe Chains

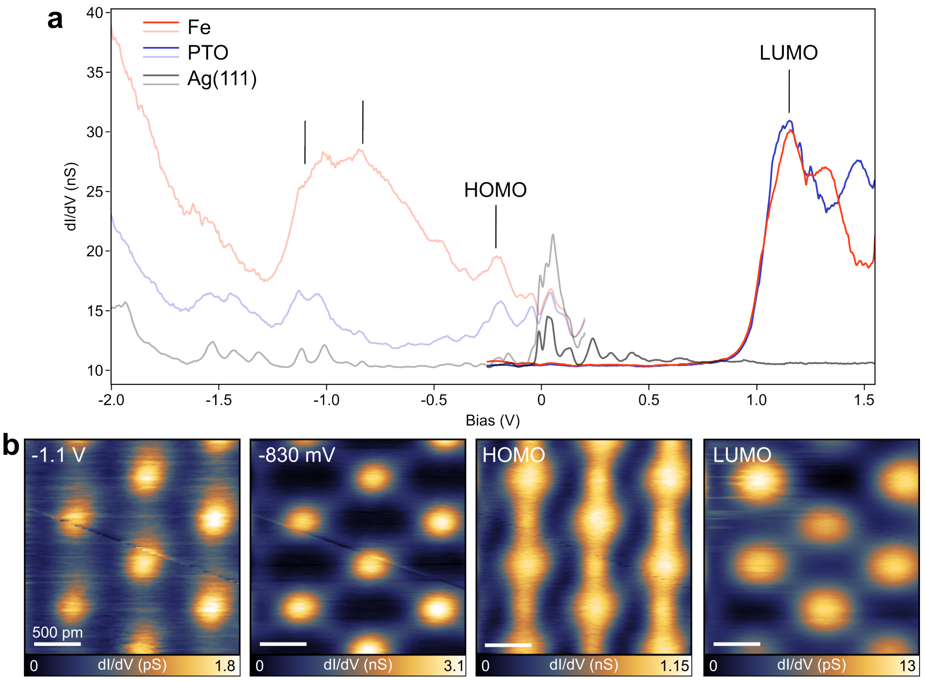

Figure S4. dI/dV maps and spectra showing molecular orbitals of PTO-Fe chains on Ag(111). **a**, dI/dV spectra measured on PTO, Fe and Ag(111) respectively. On Fe, there is pronounced DOS in the -830 mV to -1.1 V regime (LUMO: V_s = 1.1 V, I_t = 500 pA,A_{mod} = 11 mV; HOMO: V_s = 450 mV, I_t = 2 nA, A_{mod} = 11 mV). **b**, dI/dV maps of a PTO-Fe chain assembly on Ag(111) (A_{mod} = 15 mV).

The dI/dV spectrum measured with a large energy window shows frontier molecular orbitals. The PTO-Fe chain on Ag(111) (Fig. S4a) has the highest occupied molecular orbital (HOMO) at about -210 meV, and the lowest unoccupied molecular orbital (LUMO) at about 1.15 eV. Interestingly, from both the spectrum and dI/dV maps (Fig. S4b), there is a pronounced state localized on Fe in the bias range -830 mV to -1.1 V, showing a stark difference in the HOMO, which has high DOS localization on PTO molecules. In the case of PTO-Fe chains on Pb(111), molecular orbitals show similar trend as that on Ag(111). On Pb(111), HOMO locates around -100 meV and LUMO around 1.2 V. A state at about -400 meV arises exclusively on Fe, which can also be observed from dI/dV maps (Fig. S5b).

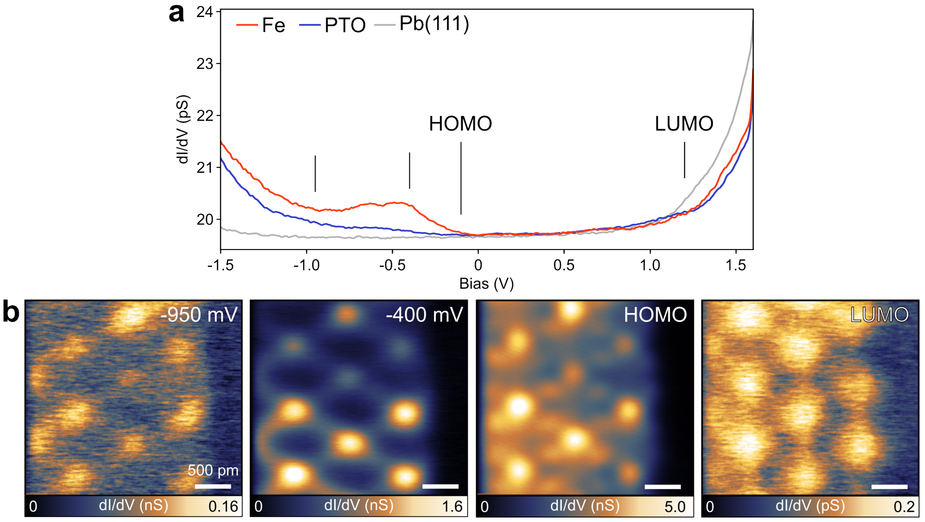

Figure S5. dI/dV maps and spectra showing molecular orbitals of PTO-Fe chains on Pb(111). **a** dI/dV spectra measured on PTO, Fe and Pb(111) respectively. Around -400 mV, there is pronounced DOS contribution from Fe (V_s = 1.55 V, I_t = 300 pA, A_{mod} = 20 mV). **b**, dI/dV maps of a PTO-Fe chain assembly on Pb(111) (A_{mod} = 20 mV).

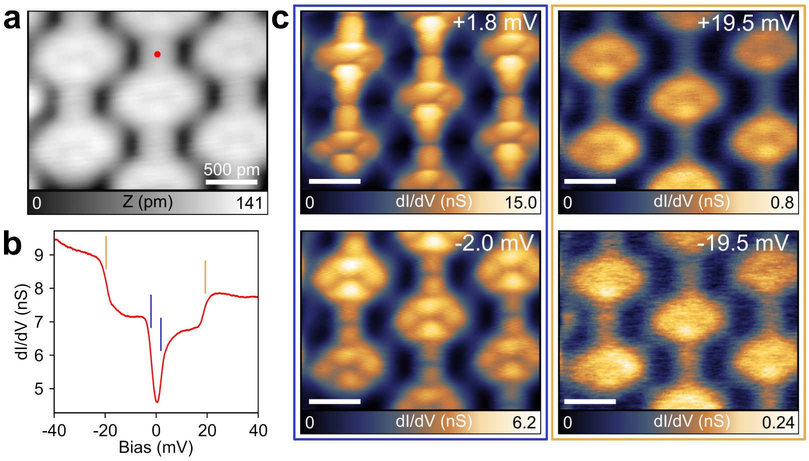

Figure S6. DOS distribution at different spin-flip excitation energies of PTO-Fe chains on Ag(111). **a**, A segment of the PTO-Fe chain assembly on Ag(111) (V_s = 50 mV, I_t = 100 pA). **b**, The dI/dV spectrum measured on Fe (red spot in a)) shows spin-flip excitations *via* two different mechanisms (V_s = 35 mV, I_t = 300 pA, A_{mod} = 1 mV). **c**, dI/dV maps of at the indicated spin-flip energies (A_{mod}= 1 mV). Scale bar: 500 pm.

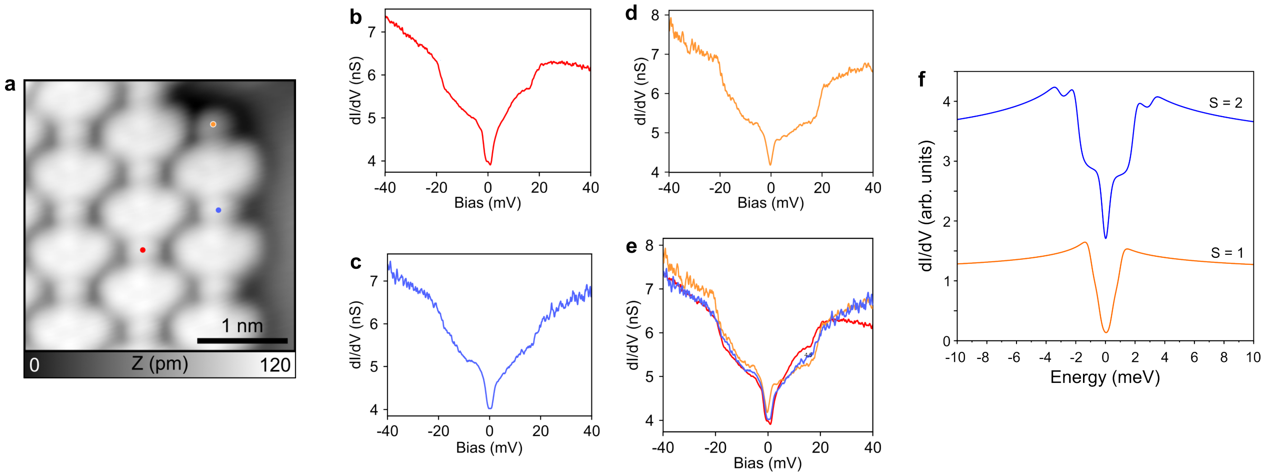

Figure S7. Comparison of tunneling spectra in and at the end of a Fe-PTO chain. **a**, Topographic STM image showing where the dI/dV spectra have been acquired (scan parameters: I_t = 100 pA, V_s = 50 mV). **b-c**, dI/dV spectra recorded at 1 K at Fe sites within Fe-PTO chains marked by a red and blue dots in **a**, (f = 613 Hz, A_{mod} = 200 µeV). **d**, dI/dV spectra at the Fe sites at the end of the Fe-PTO chain. **e**, All dI/dV spectra are superimposed in the graph. The dip at zero-energy of the dI/dV spectra obtained at the end of the chain is sharper than those in the middle of the chains whereas the steps at ±18 meV remains identical for both cases. **f**, Simulated dI/dV spectra for a S = 2 (blue) and S = 1 (orange) impurity coupled to a metal considering the parameters g = 2.11, D = -0.8 meV, E = 0.21 meV, U = 0.2, $J\rho_s$ = -0.09, T_{eff} = 1.2 K.

4. Spin excitation spectra on Pb(111)

4.1. Variation in the spin-flip excitation energies

As shown in the main manuscript, coordinated Fe in Fe-PTO chains show two pairs of spin excitations outside the superconducting gap $\pm 2\Delta$ denoted $|\varepsilon_1^*|$ and $|\varepsilon_2^*|$, respectively. These energies slightly fluctuate as a function of the considered Fe sites as shown in Figure S8. We attribute this observation to the modulation of the coupling strength with the substrate, influencing the magnetic anisotropy. We summarize in Table S1 the spin excitation energies extracted from eight spectra measured on different Fe sites on Pb(111). $|\varepsilon_\mathrm{n}|$ are defined as $|\varepsilon_\mathrm{n}| = |\varepsilon_1^*|$ - 2Δ.

Table S1. Spin excitation energies $|\varepsilon_\mathrm{n}^*|$ and $|\varepsilon_\mathrm{n}| = |\varepsilon_1^*|$ - 2Δ in meV as a function of the Fe sites on Pb(111) extracted from Fig. S8.

Fe site	$\lvert\varepsilon_1^*\rvert$	$\lvert\varepsilon_2^*\rvert$	$\lvert\varepsilon_1\rvert$	$\lvert\varepsilon_2\rvert$
**1**	4.3	4.82	1.6	2.12
**2**	4.03	4.77	1.33	2.07
**3**	3.7	4.78	1.0	2.08
**4**	3.76	4.92	1.06	2.22
**5**	3.72	4.78	1.02	2.07
**6**	4.27	4.92	1.57	2.22
**7**	3.67	4.75	0.97	2.05
**8**	4.09	4.49	1.39	1.79
Average	3.94	4.77	1.24	2.07

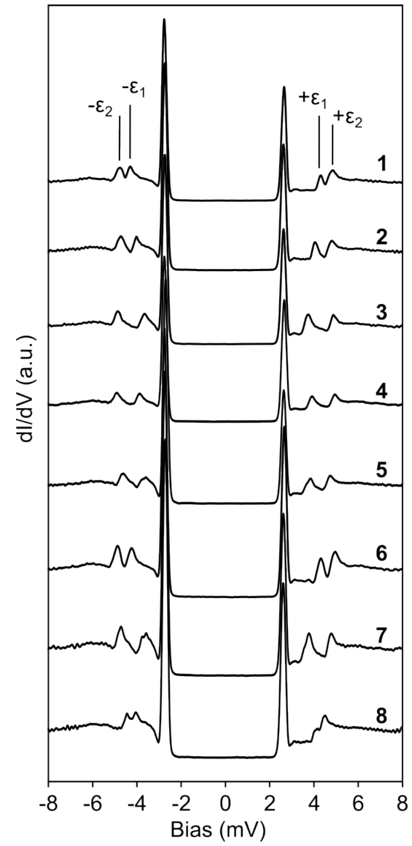

Figure S8. Spin excitations of different Fe sites along a Fe-PTO chain on Pb(111). The $\mathrm{d}I/\mathrm{d}V$ spectra are shifted for clarity. The energy of spin excitations ε_1 and ε_2 shows a slight modulation as a function of the probed Fe atoms, which values are reported in Table S1.

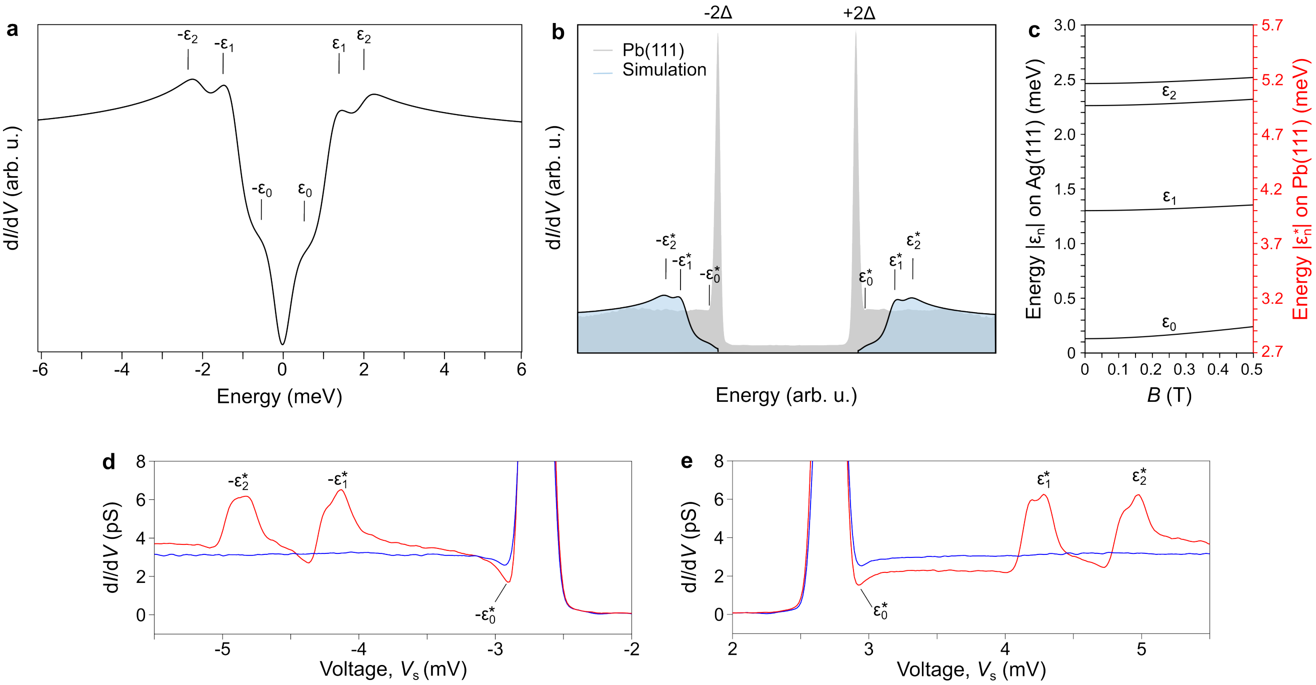

Figure S9. Simulated dI/dV spectra. **a**, Simulated dI/dV spectra for a S = 2 single impurity coupled to a metal considering g = 2.11, D = -0.55 meV, E = 0.16 meV, U = 0.2, $J\rho_s$ = -0.1, T_{eff} = 1.2 K. **b**, Schematic representation of the spin excitations steps when coupling such impurity to a superconducting substrate. **b**, Energies of the spin excitations as a function of vertical B field on Ag(111) (black axis) and on a Pb(111) by shifting by 2Δ (red). **d-e**, Zoom of the dI/dV spectra shown in Figure 3d showing the small drop of conductance at ±2.82 meV near the coherence peaks assigned to the spin excitations $\pm\,\varepsilon_0^*$.

4.2. Tunneling spectra on a Fe cluster

Coordinated PTO-Fe chains on Pb(111) show two spin excitations energies along with asymmetric coherence peaks. At a defect site (green dots in Figure S10a) which corresponds to a Fe cluster, the corresponding dI/dV of Figure S10b shows one pair of YSR states inside the superconducting gap (red arrows in Figure S10b). At the neighboring Fe inside the chain (orange dot in Figure S10a), the spin ground state and the crystal field of the iron in the PFe-PTO chain are altered leading to only one spin-flip excitation and the coexistence of YSR states (see arrows in ornage curve of Figure S10b). Although the end of the chain is deviated from the assembly (blue dot in Fig. S10a), the coordination with two PTO molecules ensures a typical two-pair spin excitations as shown in the blue spectrum in Figure¨ S10b.

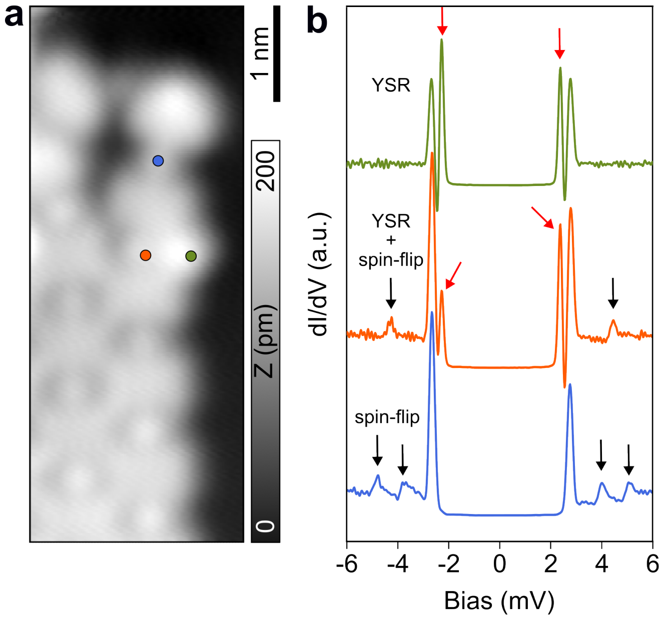

Figure S10. Coexistence of YSR in-gap states and spin excitations observed near an Fe cluster. **a**, A defect site of the PTO-Fe chain with an additional Fe atom (green dot) attached to the chain (I_t= 90 pA, V_s= 9 mV). **b**, dI/dV spectra measured at three Fe around the "defect" site of the chain. The green spectrum measured on an additional Fe shows typical YSR states without spin excitation. The orange spectrum taken at the close vicinity to the additional Fe shows the coexistence of YSR states and the spin excitation. The blue spectrum shows two pairs of spin excitation, similar to those measured in the defect-free PTO-Fe chain. Black arrows mark spin excitations and red arrows point out YSR in-gap states (I_t= 300 pA, V_s= 8 mV, A_{mod}= 50 μV).

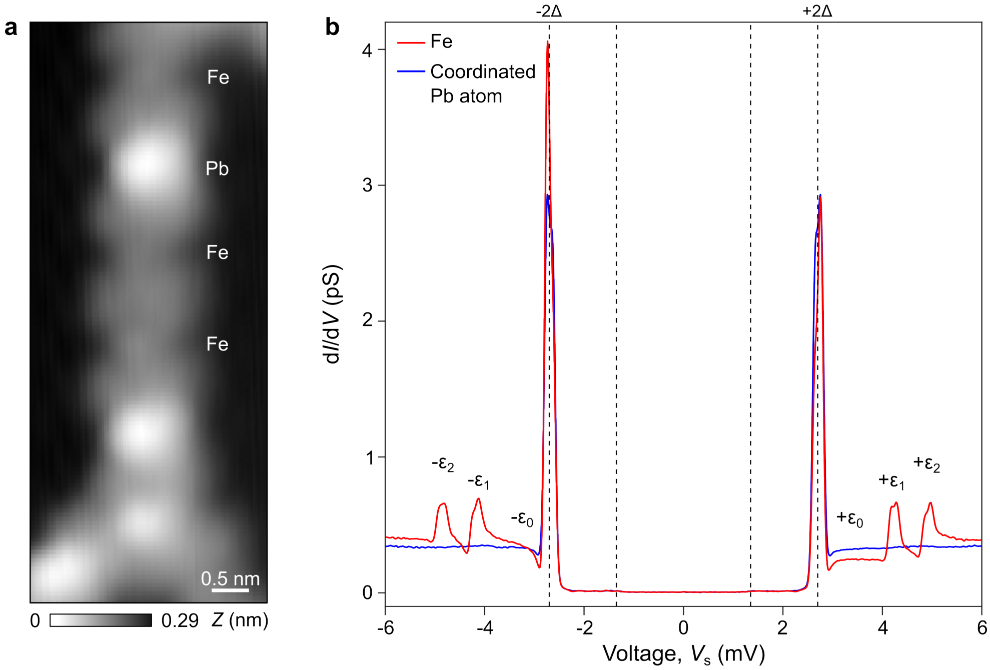

Figure S11. Absence of spin excitations for a Pb atom coordinated with PTO molecules. **a)** STM image of a Fe-PTO chain with Pb atoms incorporated in it (white protrusions named Pb) (I_t= 90 pA, V_s= 9 mV). **b)** dI/dV point-spectra measured at an Fe sites (red) as compared to one of the Pb atom (blue) (I_t= 300 pA, V_s= 8 mV, A_{mod}= 50 μV).

4.3. Spin excitations of Fe-PTO/Pb(111) under magnetic field

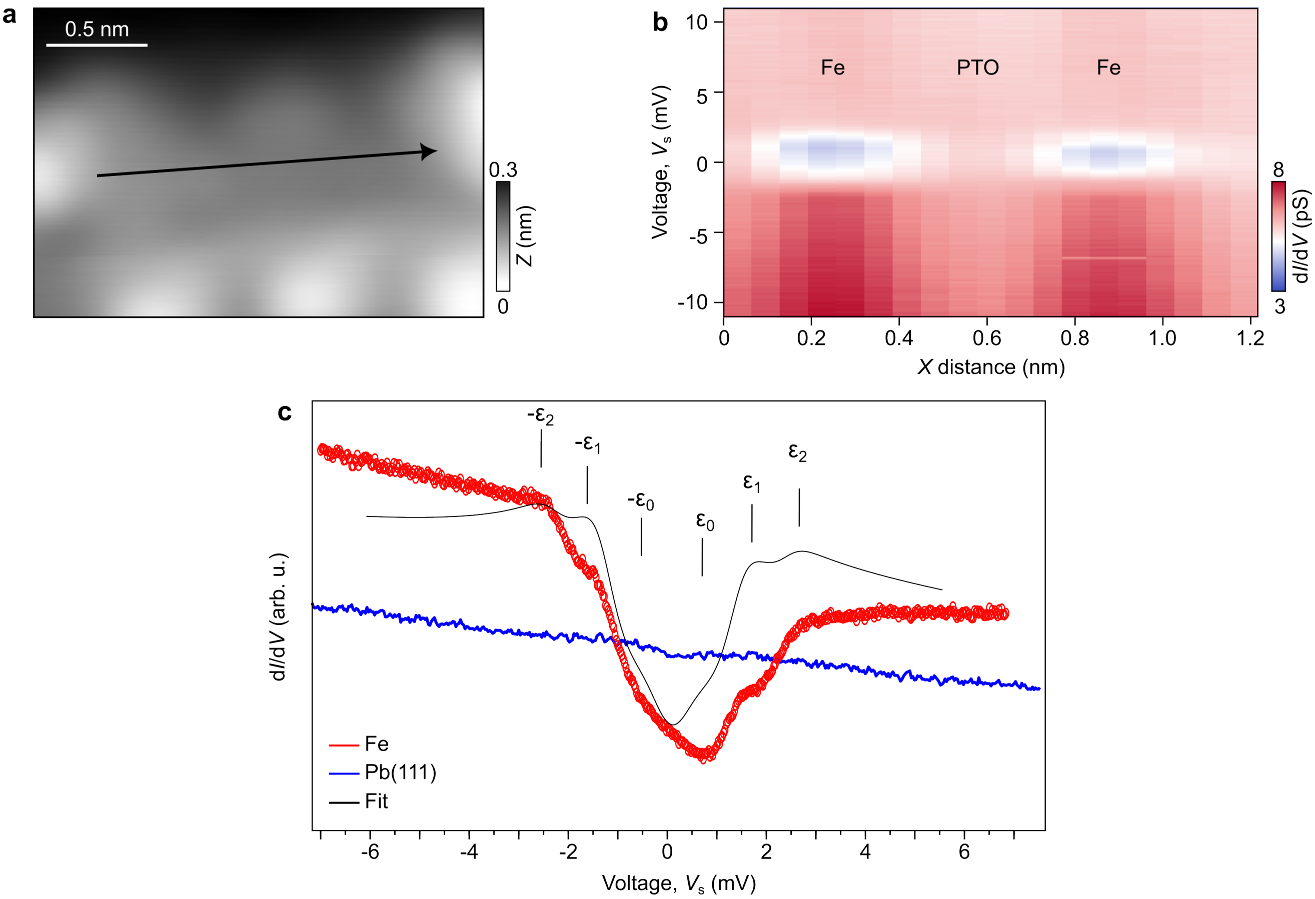

Figure S12. Spin excitations of Fe-PTO/Pb(111) under a vertical B field. **a**, STM image of the Fe-PTO chains on Pb(111) with an B field B = 0.5 T applied perpendicular to the sample, (I_t = 10 pA, V_s = 9 mV). **b**, dI/dV cross-section acquired along the arrow of **a** (I_t = 800 pA, V_s = -6 mV, A_{mod} = 20 μV). **c**, Single dI/dV point-spectra at the Fe site (red) and at the PTO molecules (blue) (I_t = 800 pA, V_s = -6 mV, A_{mod} = 600 μV). A simulated curves is shown in black with parameters g= 2, S = 2, E = -0.51, D = 0.12, B = 0.5 T.

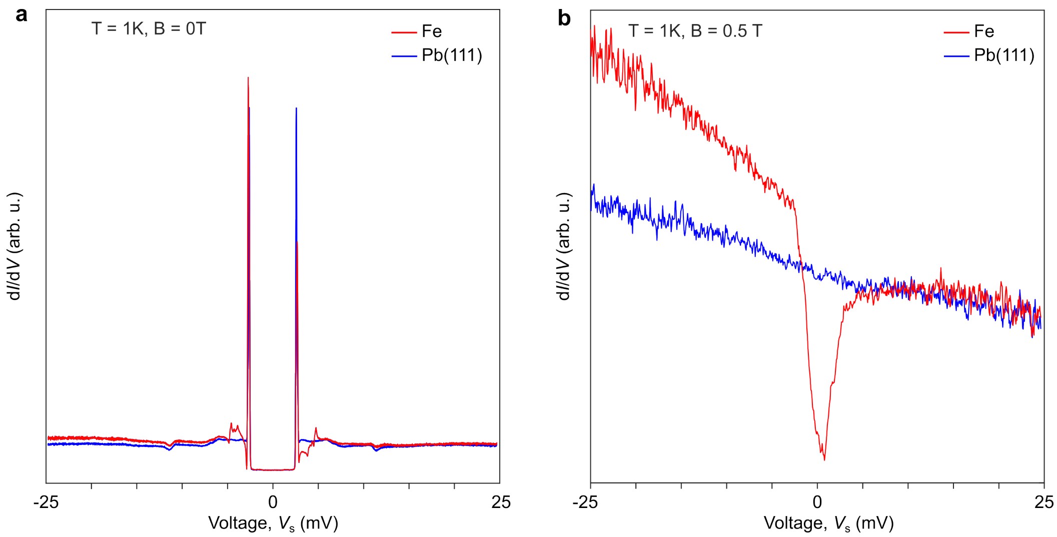

Figure S13. Absence of spin-spin excitations between neighboring Fe atoms on Pb(111). **a**, dI/dV spectra measured at a Fe site (red) and at the Pb(111) (blue) at 1 K and without B field. No spin excitations due to Fe-Fe coupling are observed (I_t = 300 pA, V_s= 8 mV, A_{mod}= 50 μV). **b**, dI/dV spectra measured at a Fe site (red) and at the Pb(111) (blue) at 1 K with an applied B field of 0.5 T. The superconducting gap is quenched by the magnetic field. No spin excitations due to Fe-Fe coupling are observed (I_t= 300 pA, V_s= 8 mV, A_{mod}= 800 μV).

References

[1] J. Hu, D. Zhang, F. W. Harris, *J. Org. Chem.* **2005**, *70*, 707.

[2] F. J. Giessibl, *Rev. Sci. Instrum.* **2019**, *90*, 011101.

[3] M. Ternes, Spin-Gui-1.0 **2015**.

[4] M. Ternes, *New J. Phys.* **2015**, *17*, 063016.

[5] J. VandeVondele, M. Krack, F. Mohamed, M. Parrinello, T. Chassaing, J. Hutter, *Computer Physics Communications* **2005**, *167*, 103.

[6] T. D. Kühne, M. Iannuzzi, M. Del Ben, V. V. Rybkin, P. Seewald, F. Stein, T. Laino, R. Z. Khaliullin, O. Schütt, F. Schiffmann, D. Golze, J. Wilhelm, S. Chulkov, M. H. Bani-Hashemian, V. Weber, U. Borštnik, M. Taillefumier, A. S. Jakobovits, A. Lazzaro, H. Pabst, T. Müller, R. Schade, M. Guidon, S. Andermatt, N. Holmberg, G. K. Schenter, A. Hehn, A. Bussy, F. Belleflamme, G. Tabacchi, A. Glöß, M. Lass, I. Bethune, C. J. Mundy, C. Plessl, M. Watkins, J. VandeVondele, M. Krack, J. Hutter, *J. Chem. Phys.* **2020**, *152*, 194103.

[7] J. P. Perdew, K. Burke, M. Ernzerhof, *Phys. Rev. Lett.* **1996**, *77*, 3865.

[8] S. Grimme, J. Antony, S. Ehrlich, H. Krieg, *The Journal of Chemical Physics* **2010**, *132*, 154104.

[9] V. I. Anisimov, J. Zaanen, O. K. Andersen, *Physical Review B* **1991**, *44*, 943.

[10] S. L. Dudarev, G. A. Botton, S. Y. Savrasov, C. J. Humphreys, A. P. Sutton, *Phys. Rev. B* **1998**, *57*, 1505.

[11] S. Goedecker, M. Teter, J. Hutter, *Phys. Rev. B* **1996**, *54*, 1703.

[12] J. VandeVondele, J. Hutter, *J. Chem. Phys.* **2007**, *127*, 114105.

[13] J. VandeVondele, J. Hutter, *J. Chem. Phys.* **2003**, *118*, 4365.

[14] L. Bengtsson, *Phys. Rev. B* **1999**, *59*, 12301.
